# A bibliometric review and visual analysis of orthotic treatment in adolescent idiopathic scoliosis from the Web of Science database and CiteSpace software

**DOI:** 10.1097/MD.0000000000036958

**Published:** 2024-01-12

**Authors:** Changliang Luo, Huidong Wu, Wei Liu, Mansang Wong

**Affiliations:** a Department of Biomedical Engineering, The Hong Kong Polytechnic University, Hong Kong SAR, China; b Department of Prosthetic and Orthotic Engineering, School of Rehabilitation, Kunming Medical University, Kunming, China.

**Keywords:** advancement, AIS, bibliometric analysis, orthosis, research trend

## Abstract

Orthotic treatment has been the primary nonoperative treatment for patients with adolescent idiopathic scoliosis (AIS), however, no bibliometric study has been conducted in this field to date. Therefore, this study aims to analyze potential trends and new advances in the field of orthotic treatment of AIS through a bibliometric analysis and visualization study. Relevant literature included in the Web of Science database from the start of the database to the 1st month of 2023 was retrieved and analyzed using CiteSpace software (version 6.1.R6). Data on the nations, institutions, authors, journals, keywords, and cited references were collected for each publication. A total of 1005 records were included. The most productive countries and institutions were the USA and Hong Kong Polytechnic University, respectively. *Spine* was the most influential journal, with the highest number of citations. Hubert Labelle had the most publications, whereas Weinstein was the most cited author. The efficacy of orthotic treatment has always been at the frontier of research. Notably, changes in the quality of life after orthotic treatment, success rate or curve progression, new classification systems, and exercises have been the focus of research in recent years. This study enriches the understanding of research landscapes and key contributors in orthotic treatment for AIS.

## 1. Introduction

Scoliosis is a complicated 3-dimensional spine deformity which usually involves a lateral curvature in the coronal plane and an axial rotation in the horizontal plane.^[1]^ Abnormal alignment in the sagittal plane, such as kyphosis and lordosis may also be observed in some cases.^[2]^ Adolescent idiopathic scoliosis (AIS) is the most typical type that affects 1% to 3% of young teenagers, and progresses during the rapid growth period from age 10 to 16 years with undefined causes.^[3]^

Depending on the severity of spinal curvature and the potential for further growth, treatment options for AIS include observation, orthotic management, exercise, and surgery.^[3,4]^ Orthotic treatment is one of the most effective conservative treatments, and has been shown to halt the progression of curves and avoid the subsequent need for surgery.^[5]^ Various studies have been conducted on the orthotic treatment of AIS. However, it is difficult for scholars and clinicians to identify the latest research directions and new developments as there are numerous studies on this topic.

In recent years, the bibliometric analysis has grown in popularity by employing literary metrology features to evaluate the contribution of a research field, including different countries, institutions, publications, and authors. Additionally, it helps to forecast research trends or hotspots on a certain topic. To the best of our knowledge, no bibliometric studies have been conducted in the field of orthotic treatment of AIS.

CiteSpace is a visual analysis software based on the JAVA language environment, which is now widely used in many fields, such as information science, education, and medicine.^[6–8]^ The use of CiteSpace software for bibliometric and visualization analysis could assist researchers in comprehending research status, hotspots, and tendencies in a specific field, as well as provide good references for their research. The core collection database of the Web of Science (WOS) contains over 12,000 excellent quality journals from various nations worldwide. The WOS is one of the most comprehensive, systematic, and authoritative databases and is one of the most popular databases for scientometric evaluation with complete references and citations.^[9,10]^ Therefore, this study utilized the WOS database for the literature search and employed CiteSpace software to analyze the records. Our findings aim to begin to build a knowledge map of this field and allow scholars and clinicians to better understand the advancement and future directions of this treatment to develop new schemes for higher treatment success rates and to provide better services.

## 2. Methods

### 2.1. Sources of data and search strategy

Relevant literature on the orthotic treatment of AIS published from January 1, 1985, to January 28, 2023, was comprehensively searched utilizing the WOS database. The following search strategies were used: all fields with (“adolescent idiopathic scoliosis” OR “AIS” OR “idiopathic scoliosis”) AND (“brace” OR “bracing” OR “orthosis” OR “orthoses” OR “orthotic”), without limitations on language. Records were obtained on the same day (January 28, 2023) to avoid potential bias.

### 2.2. Literature selection criteria

Original articles, reviews, and conference papers were selected for review. Other document types such as news, letters, and bulletins were excluded. The retrieved literature was then exported into a plain text document format with the contents including “full records and cited references.” Every 500 records were exported to a file named “download_.txt.” and saved to a specially created folder named “input.” A total of 1005 records were retrieved and saved as 3 files in the same folder. The title, authors, abstract, keywords, and reference lists were included in each record.

### 2.3. Parameter setting in CiteSpace software

CiteSpace software (Version 6.1.R6, Drexel University, Philadelphia, USA) was used to conduct this analysis and to procure collaborative networks (e.g., authors, countries, and institutions), impact networks (e.g., co-cited references, co-cited authors, and co-cited journals), and co-occurrence keyword analyses. In the parameter configurations, the time span was set from January 1985 to January 2023, the time slice was set to 1 year, and in the text processing function zone, abstract, title, author keywords, and keywords plus were chosen as the term source; the term type included both noun phrases and burst terms. The node types were chosen according to the different purposes of the analysis. Authors, countries, institutions, keywords, references, cited authors, and cited journals were selected in sequence for co-occurrence and co-citation visualization analysis and to draw the network graphs. In the link part, the strength was set as cosine and the scope was within the slices. Then “Top N” was selected in the threshold item, and the value was set to 50. “Pathfinder” and “Pruning sliced networks” were picked as cut connection methods to make the network structure simpler and emphasize key features.

### 2.4. Study design

This bibliometric review was conducted using the CiteSpace software by analyzing the following content: Collaborating institutions and countries in this field. When “institution” and “country” in the node type of the parameter configurations were chosen, the results about the publication and collaborating information were shown in tables and knowledge graphs. In the knowledge graph, the thickness of the link and the distance between nodes reveal the degree of collaboration across countries, organizations, and authors. The link between nodes represents a co-occurrence or co-citation relationship.^[11]^ Moreover, the betweenness centrality of a network node quantifies the significance of the node position within the network, and the distribution of journal sources. The “cited journal” should be selected in the node type of the settings. Each node size reflects the frequency of citations or the degree of co-occurrence. The authors and co-cited authors. The “author” and “cited author” were chosen as the node type. By analyzing the publications and citations of authors, information related to the main research personnel and their teamwork in the area was provided. The keyword group that combines phrases and words. “keyword” and “term” were selected, then the software would automatically analyze the keywords with obvious field properties into clustering targets, employ original feature extraction algorithms for text classification to perform word domain clustering, and regulate the impact of word frequency to obtain general and specific domain words. A cluster analysis of keywords was performed in this review, and the silhouette function was employed to evaluate clusters. A silhouette value (S) >0.7, indicated that the cluster members were highly homogeneous and that the clustering results were valid. If it was >0.5, clustering was typically considered acceptable.^[9]^ Another structural metric used to determine cluster quality is modularity.^[7]^ The modularity score (Q) indicates the extent to which a network can be divided into modules.^[12]^ The Q score varies from 0 to 1 and is deemed reasonable if it exceeds 0.3. The higher the modularity value, the more effectively the network is constructed. In addition, bursts of keywords were extracted to trace the evolution of research emphasis. A burst of keywords refers to frequently cited keywords over a specific time period, which are essential for determining the frontier subjects and key areas of research^[13]^; the most frequently cited publications. The “reference” was selected as the node type. In the graph, the lines represent the connections between the nodes, and their colors indicate the years of publication.^[14]^ The centrality of a node typically reflects whether the node is an important turning point or a pivot in a domain.^[15]^ Owing to their groundbreaking contributions, the most frequently cited publications are typically recognized as milestones.

## 3. Results

### 3.1. The output of related publications

**Figure 1.**
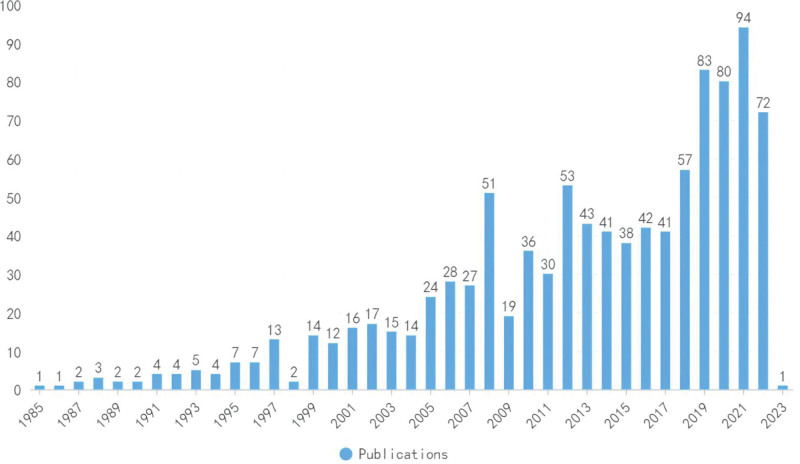
Publication outputs over the yr. The overall publication outputs have shown an upward trend, which reflected the increasing amount of attention given to the orthotic treatment for the patients with AIS by scholars. AIS = adolescent idiopathic scoliosis.

A total of 1005 records were retrieved, including 832 original articles, 102 reviews, and 71 conference papers. Although there have been minor fluctuations since 1985, the overall publication output has shown an upward trend, which reflects the increasing amount of attention given to orthotic treatment for patients with AIS. The highest number of papers was published in 2021, with 94 publications. The upward trend in the last 5 years was obvious, indicating an increasing interest from researchers (Fig. 1). However, the potential decreasing trend from 2021 may also indicate that the subsequent development force was insufficient, and experts in this area should pay more attention to enlarging and intensifying the research foundation to maintain a steady development trend.

### 3.2. Visual analyses of countries and institutions

**Figure 2.**
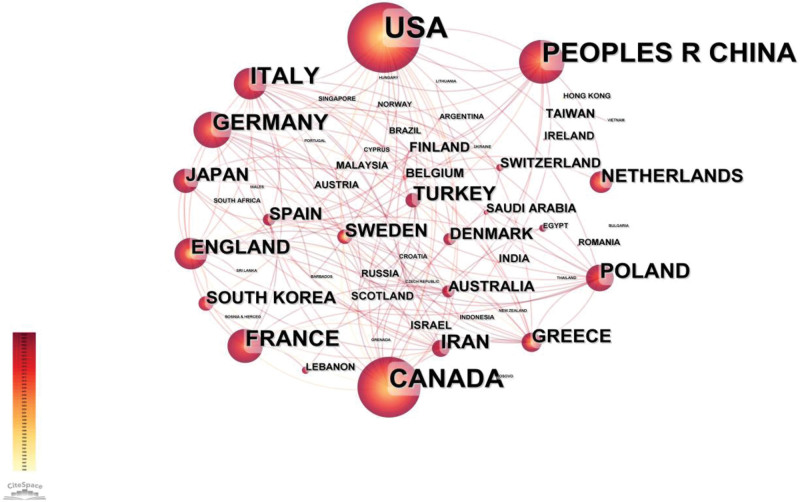
The network map of productive countries/regions (the nodes on the map represent countries or regions, while the lines linking them represent co-citation associations. The different colors of the nodes signify distinct yr).

**Figure 3.**
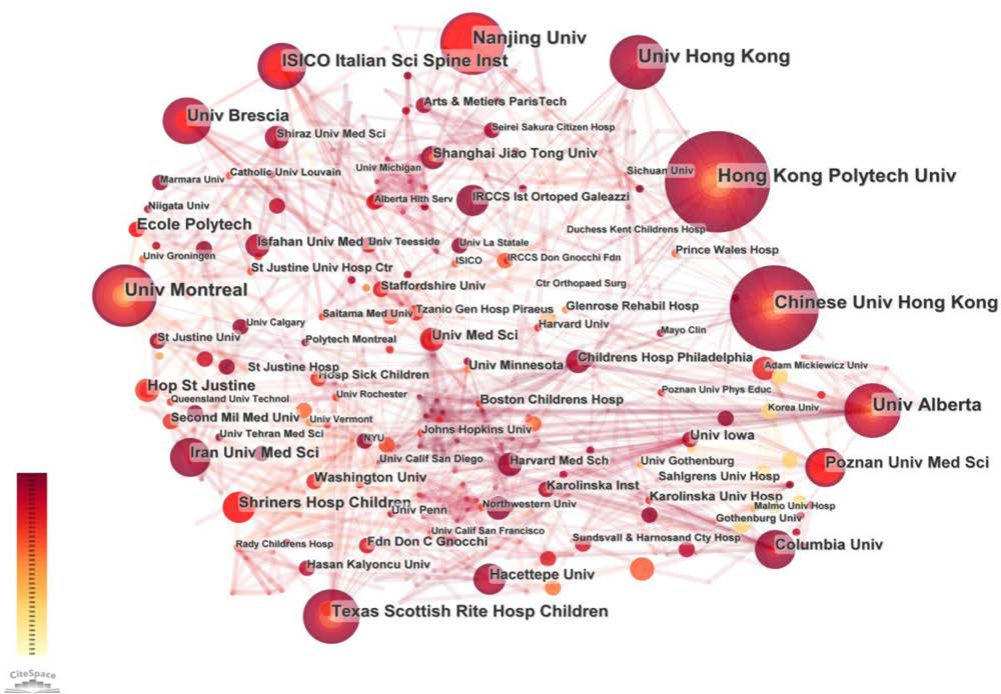
The network map of institutions (greater the node area, greater the quantity of publications).

The collaborating countries in this field were analyzed, and 57 countries or regions published papers related to orthotic intervention in AIS (Fig. 2). There were 20 countries or regions with more than 10 papers, and the country with the most published articles was the United States, with 253 publications, reflecting its dominant position in this field. China ranked second with 159 publications, followed by Canada with 126 publications, Italy with 72 publications, France with 57 publications, and Germany with 55 publications (Table 1). An analysis of the institutions can reveal important research forces in the field and the characteristics of the activities of each research institution. The analysis included 1204 institutions (Fig. 3). The top 6 institutions in terms of published work were The Hong Kong Polytechnic University (n = 38), University of Hong Kong (n = 35), Chinese University of Hong Kong (n = 33), University of Montreal (n = 33), Nanjing University (n = 30), and University of Alberta (n = 30). Of the top 6 institutions, the first 3 were in Hong Kong and China, the fifth was in Mainland China, and the other 2 were in Canada (Table 1).

**Table 1:** Top 6 high-productive countries and institutions.

Rank	Countries	Publications	Rank	Institutions	Publications
1	USA	253	1	The Hong Kong Polytechnic University	38
2	People Republic Of China	159	2	The University of Hong Kong	35
3	Canada	126	3	The Chinese University of Hong Kong	33
4	Italy	72	4	University of Montreal	33
5	France	57	5	Nanjing University	30
6	Germany	55	6	University of Alberta	30

### 3.3. Visual analyses of co-cited journals

**Figure 4.**
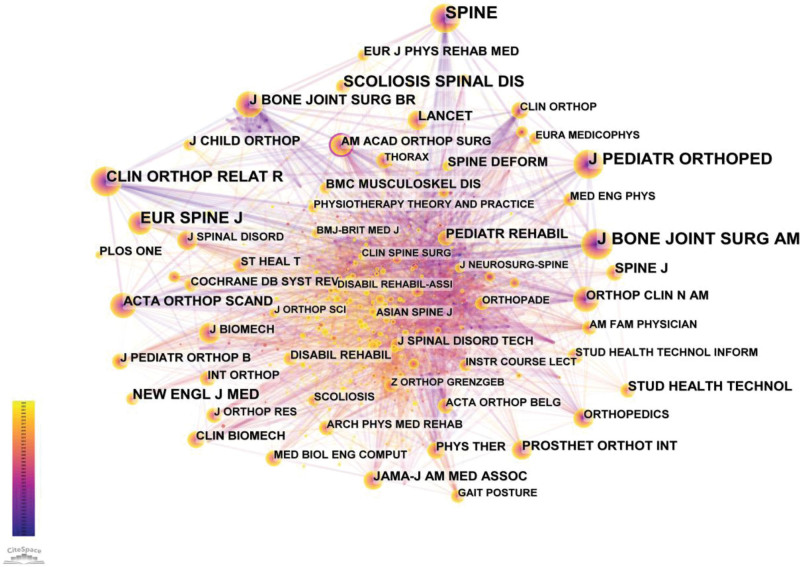
The network map of co-cited journals.

Analyzing the distribution of journal sources can assist researchers in quickly selecting the most suitable journals for their papers.^[16]^ The 1005 publications related to spinal orthoses for AIS were co-cited by a total of 1042 journals (Fig. 4). Spine was the most frequently cited journal, with 922 citations, followed by the Journal of Bone and Joint Surgery-American Volume (751 citations), European Spine Journal (646 citations), Journal of Pediatric Orthopaedics (548 citations), and Scoliosis and Spinal Disorders (476 citations). The top 5 journals in terms of citations and centrality are listed in Table 2.

**Table 2:** Top 5 journals in terms of citation and centrality.

Rank	Journal	Citation	Rank	Journal	Centrality
1	Spine	922	1	Acta Psychiatrica Scandinavica	0.14
2	the Journal of Bone and Joint Surgery—American Volume	751	2	Proceedings Of The National Academy Of Sciences Of The United States Of America	0.1
3	European Spine Journal	646	3	Archives Of Physical Medicine And Rehabilitation	0.1
4	Journal of Pediatric Orthopaedics	548	4	Acta Orthopaedica Belgica	0.09
5	Scoliosis and Spinal Disorders	476	5	Journal of the American Academy of Orthopaedic Surgeons	0.09

### 3.4. Visual analyses of authors and cited authors

**Figure 5.**
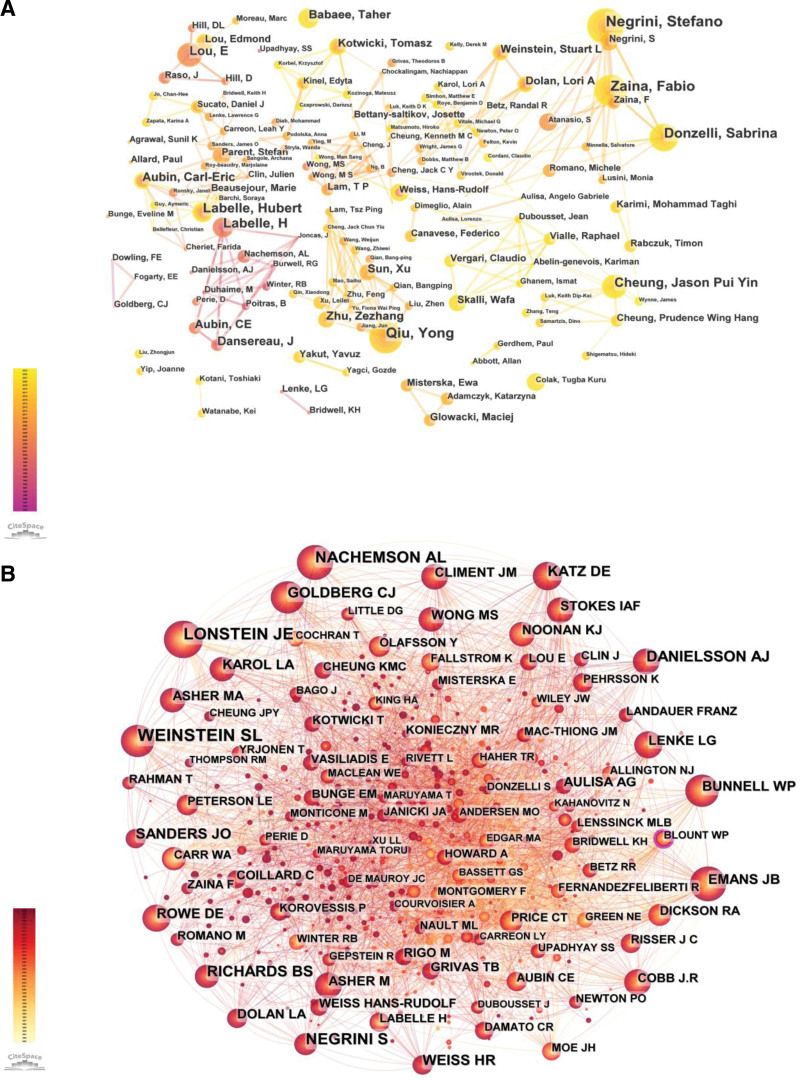
The network map of productive authors (A) and co-cited authors (B).

Information on the authors (Fig. 5A) and co-cited authors (Fig. 5B) was extracted and visualized in a knowledge domain map using CiteSpace. The 818 authors who published more than 15 papers in this field, and those who were cited more than 100 times, are listed in Table 3.

**Table 3:** Authors with publications ≥ 15 and with citations ≥ 100.

Rank	Author	Publications	Rank	Cited author	Citations
1	Labelle H	45	1	Weinstein SL	443
2	Negrini S	40	2	Lonstein JE	319
3	Zaina F	34	3	Negrini S	283
4	Lou E	33	4	Nachemson AL	246
5	Hill D	29	5	Danielsson AJ	225
6	Qiu Y	27	6	Richards BS	199
7	Aubin CE	26	7	Katz DE	189
8	Cheng JCY	25	8	Goldberg CJ	159
9	Wong MS	25	9	Weiss HR	151
10	Cheung JPY	24	10	Sanders JO	150
11	Raso J	23	11	Bunnell WP	146
12	Donzelli S	22	12	Emans JB	141
13	Zhu ZZ	21	13	Karol LA	137
14	Sun Xu	18	14	Rowe DE	120
15	Lam TP	17	15	Wong MS	115
16	Danielsson A	16	16	Lenke LG	111
17	Kotwicki T	16	17	Asher M	110
18	Moreau M	16	18	Dolan LA	109
19	Romano M	16	19	Climent JM	106
20	Cheung PWH	15	20	Asher MA	104
21	Dansereau J	15	21	Aulisa AG	104
22	Dolan LA	15	22	Noonan KJ	100
23	Weinstein SL	15	23	Stokes IAF	100

Labelle Hubert, from the Division of Orthopaedic Surgery at the University of Montreal, Canada, had the most publications (n = 45). The second was Negrini Stefano with 40 publications from the Department of Clinical and Experimental Sciences at the University of Brescia, Italy. The third was Zaina Fabio with 34 publications from the Italian Scientific Spine Institute. Edmond Lou, from the University of Alberta in Canada, ranked fourth with 33 publications, and Doug Hill ranked fifth with 29 publications from the Glenrose Rehabilitation Hospital in Canada.

Additionally, Weinstein (443 citations) ranked first among the top co-cited authors who contributed to publications in research on AIS orthotic treatment, followed by Lonstein (319 citations), Negrini (283 citations), Nachemson (246 citations), and Danielsson (225 citations) (Table 3), demonstrating that they were influential scholars in this field and have conducted extensive research to lay a solid foundation.

The network map of authors reveals a variety of collaborative relationships among the authors (Fig. 5A). There were 5 large research teams, with Negrini Stefano, Labelle Hubert, Qiu Yong, Cheung Jason Pui Yin, and Lou Edmond as the core members of different teams, which have made great contributions and established authority in the research direction of the orthotic treatment of AIS. Some of these teams also have close connections, which is a good sign of a collaborative relationship in this field.

### 3.5. Visual analyses of keywords

#### 3.5.1. Clusters of keywords.

**Figure 6.**
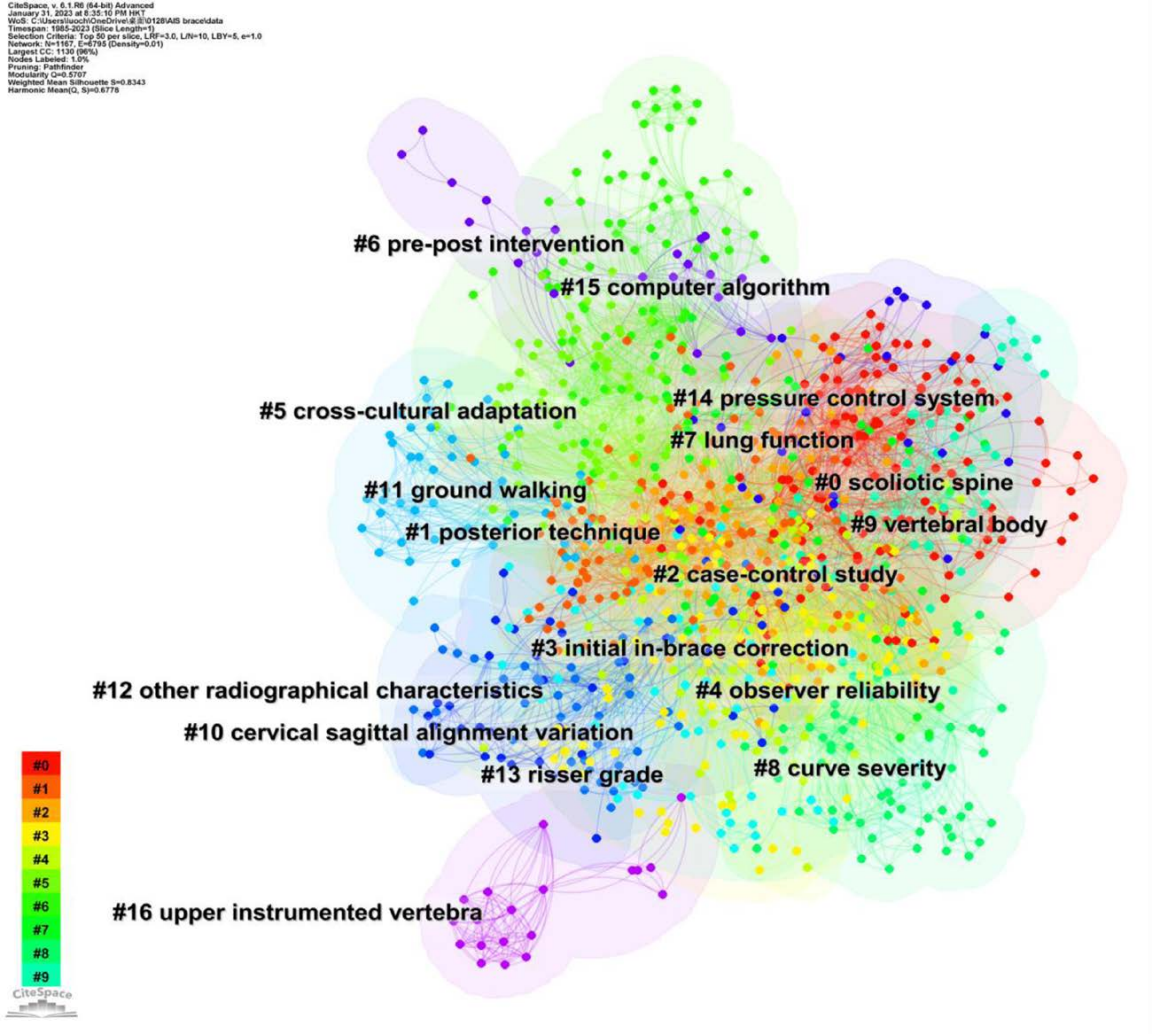
The network map of keywords clusters.

The classical ratio (log-likelihood ratio) algorithm was applied to obtain 17 major keyword cluster groups (Table 4 and Fig. 6). Cluster quality is reflected in the silhouette (S) and modularity (Q) scores. The S-score of clustering was 0.8343 > 0.7, and the Q score was 0.5707 > 0.3, indicating that the cluster members were highly consistent and that the results of the analysis were credible.

**Table 4:** The largest 17 keywords clusters.

Cluster	Label (LLR)	Size	Silhouette	Cluster members
#0	Scoliotic spine	149	0.77	Adolescent idiopathic scoliosis; Boston brace; Milwaukee brace
#1	Posterior technique	101	0.796	Idiopathic scoliosis; Charleston bending brace; fusion
#2	Case-control study	100	0.765	Quality of life; curve progression; follow up
#3	Initial in-brace correction	92	0.789	Progression; conservative treatment; Cobb angle
#4	Observer reliability	89	0.785	Children; prediction; system
#5	Cross-cultural adaptation	86	0.81	Reliability; validity; questionnaire
#6	Pre-post intervention	75	0.893	Pattern; Scheuermann kyphosis; spinal orthosis
#7	Lung function	63	0.859	Brace; adolescent; exercise
#8	Curve severity	60	0.89	TLSO; association; height
#9	Vertebral body	60	0.866	Girl; brace treatment; research society
#10	Cervical sagittal alignment variation	54	0.863	Spinal deformity; term follow up; classification
#11	Grounding walking	49	0.946	Back pain; outcome; low back pain
#12	Other radiographical characteristics	43	0.9	Instrumentation; syringomyelia; kyphosis
#13	Risser grade	39	0.881	Growth; curve; severity
#14	Pressure control system	28	0.897	Criteria; orthosis; quality
#15	Computer algorithm	23	0.973	Spinal orthoses; fusion level; CAD/CAM method
#16	Upper instrumented vertebra	19	0.991	Clinical outcome; cartilage; degeneration

CAD/CAM = computer aided design and manufacturing, LLR = log-likelihood ratio, TLSO = Thoraco-Lumbo-Sacral Orthosis.

With the exception of the cluster #2 case-control study, which was about the study type in this field, cluster #1, #12, and #16 were all about the surgical treatment of AIS. Current research about spinal orthoses of AIS primarily focuses on: the effect of orthotic treatment on physical structures and functions, such as cluster #0 scoliotic spine, #3 initial in-brace correction, #7 lung function, #8 curve severity, #9 vertebral body, and #11 grounding walking; the design of orthoses, such as #14 pressure control system, and #15 computer algorithm; the assessment of scoliosis, such as #4 observer reliability, #5 cross-cultural adaptation, #6 pre-post intervention, #10 cervical sagittal alignment variation, and #13 Risser grade.

#### 3.5.2. Burstness of keywords.

**Figure 7.**
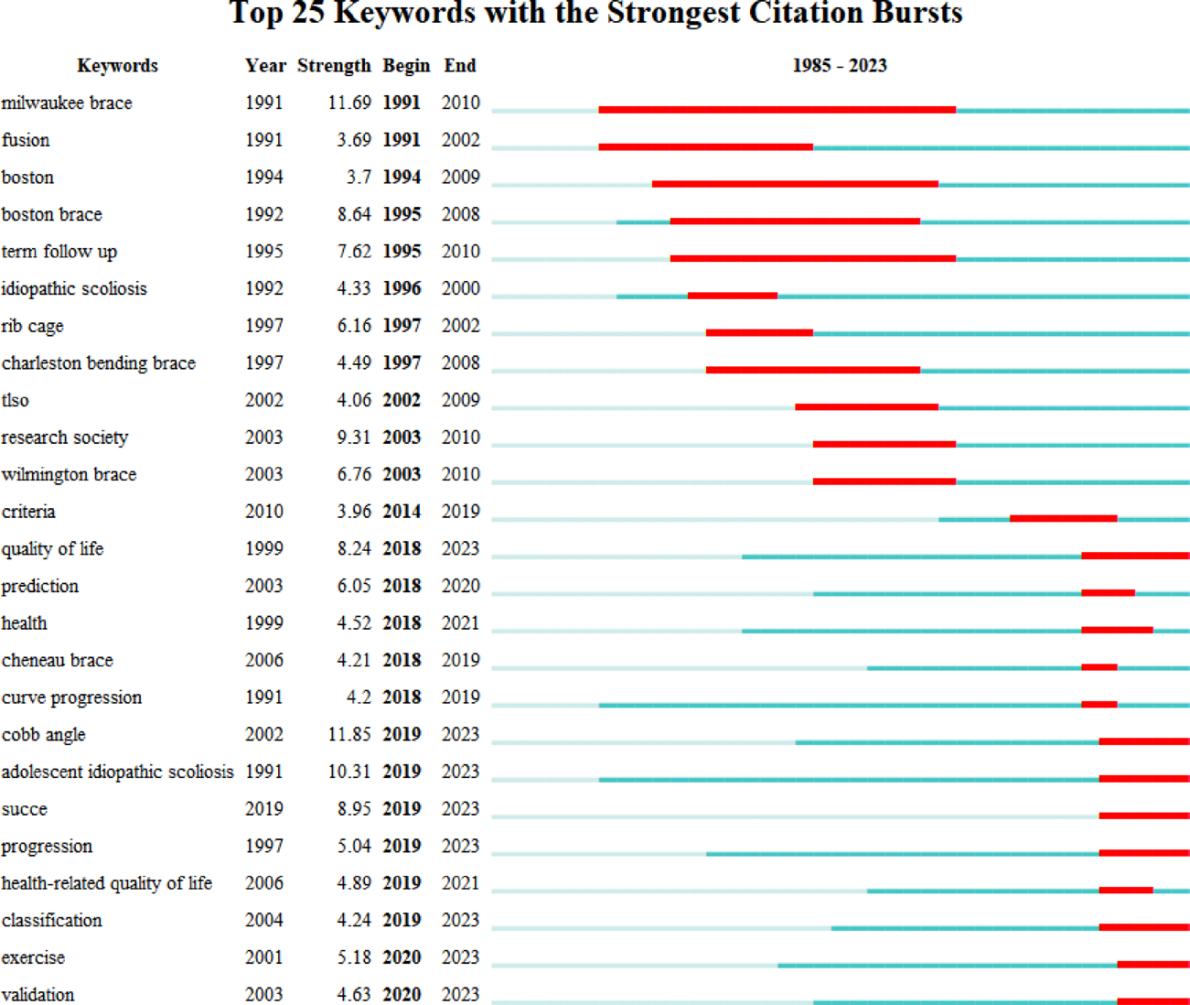
Top 25 keywords with the strongest citation bursts (the bars with red color indicated that the term was cited frequently, while the green bars indicated that the keyword was cited less frequently).

The top 25 keywords with the most powerful citation bursts are shown in Figure 7. The “Cobb angle” was the keyword with the most intense mutation (strength was 11.85), the second was “Milwaukee brace” (strength was 11.69), and “adolescent idiopathic scoliosis” was in the third place (strength was 10.31). The types of spinal orthoses, such as “Milwaukee brace,” “Boston brace,” “Charleston bending brace,” “Wilmington brace,” “Cheneau brace,” and “Thoraco-Lumbo-Sacral Orthosis”, have been a research center for a long time. Among these types of orthoses, the Milwaukee brace has attracted the interest of researchers for the longest time period, from 1991 to 2010. Research on the burst words of “quality of life,” “Cobb angle,” “success,” “progression,” “classification,” “exercise,” and “validation” began 5 years ago and has continued until now. Furthermore, it is likely to be the research focus in the coming years.

As demonstrated in the keyword analysis, co-occurring keywords and developing trends vary continuously over time, indicating that the topics and directions of the studies change correspondingly. Researchers need to keep looking into and analyzing the research trends of orthotic treatment for AIS so that they can maintain an up-to-date understanding and have a competitive edge in this field.

### 3.6. Visual analyses of co-cited references

In total, 2621 references were cited in the WOS database on the topic of orthotic treatment for AIS. Detailed information on the top 5 publications sorted by citation frequency and centrality is presented in Table 5.

**Table 5:** Top 5 cited references in terms of citation frequency and centrality.

Rank	Author	Yr	Title	Citation frequency
1	Weinstein SL	2013	Effects of Bracing in Adolescents with Idiopathic Scoliosis	98
2	Negrini S	2018	2016 SOSORT Guidelines: Orthopedic and Rehabilitation Treatment of Idiopathic Scoliosis During Growth	70
3	Karol LA	2016	Effect of Compliance Counseling on Brace Use and Success in Patients with Adolescent Idiopathic Scoliosis	34
4	Thompson RM	2017	Brace Success is Related to Curve Type in Patients with Adolescent Idiopathic Scoliosis	33
5	Negrini S	2012	2011 SOSORT Guidelines: Orthopaedic and Rehabilitation Treatment of Idiopathic Scoliosis During Growth	30
Rank	Author	Yr	Title	Centrality
1	Nicholson GP	2003	The Objective Measurement of Spinal Orthosis Use for the Treatment of Adolescent Idiopathic Scoliosis	0.17
2	Danielsson AJ	2007	A Prospective Study of Brace Treatment Versus Observation Alone in Adolescent Idiopathic Scoliosis: A Follow-up Mean of 16 Yr After Maturity	0.17
3	Qiu Y	2008	Bone Mineral Accrual in Osteopenic and Nonosteopenic Girls With Idiopathic Scoliosis During Bracing Treatment	0.11
4	Havey R	2002	A Reliable and Accurate Method for Measuring Orthosis Wearing Time	0.1
5	Janicki JA	2007	A Comparison of the Thoracolumbosacral Orthoses and Providence Orthosis in the Treatment of Adolescent Idiopathic Scoliosis-Results Using the New SRS Inclusion and Assessment Criteria for Bracing Studies	0.1

SOSORT = The International Scientific Society on Scoliosis Orthopaedic and Rehabilitation Treatment.

The research in these top articles with the most citations and the highest centrality was mostly about the effectiveness and success rate of orthotic treatment,^[5,17–20]^ the International Scientific Society on Scoliosis Orthopaedic and Rehabilitation Treatment guidelines,^[21,22]^ growth potential,^[23]^ patient compliance,^[19,24,25]^ and Scoliosis Research Society (SRS) research criteria.^[18]^

## 4. Discussion

### 4.1. Summary of the findings

The main objective of this bibliometric review was to analyze the potential trends and new advances in the field of orthotic treatment of AIS. The results indicated that the overall publication output in this area showed an upward trend. In addition, the efficacy and effectiveness of orthotic treatments have always been the focus of research. Changes in the quality of life after orthotic treatment, success rate or curve progression, new classification systems, and exercises have been researched in recent years. The application of computer algorithms, novel designs of spinal orthoses, and pressure control systems are new advancements that can be implemented. Furthermore, the results revealed that the most productive countries and institutions were the USA and Hong Kong Polytechnic University. *Spine* was the most influential journal with the most citations, Labelle Hubert had the most publications, and Weinstein SL was the most cited author. This information could be helpful for freshmen in this field to rapidly search for and understand orthotic treatment of AIS.

### 4.2. Research foundations and emerging trends

Analyses of keyword clusters and burstness can identify popular topics and trends in this field during different periods. According to keyword cluster markers, scholars and experts have paid significant attention to investigating the effect of orthotic treatment on physical structures and functions. Initial in-orthosis correction is one of the most important parameters that can be used to predict the final outcome of orthotic treatment, and an increasing number of studies have been conducted to explore the cutoff value of initial in-orthosis correction or to validate prediction models based on initial in-orthosis correction.^[26,27]^ Peeters et al also reported that increased curve flexibility is a significant predictive factor for in-orthosis correction.^[28]^ Therefore, finding a method to obtain higher curve flexibility and better in-orthosis correction is a direction for future research.

According to the burstness of keywords, research on quality of life has been a hot topic since 2018, when Meng et al conducted a study to use the SRS-22 questionnaire to measure the quality of life of patients with AIS under orthoses treated and untreated, and found that patients who received orthotic treatment had a higher quality of life.^[29]^ Subgroup analyses of QOL and its influencing factors are also of interest. Moreover, research emphasis on orthotic treatment for AIS has changed over time. Different types of orthoses have been a research hotspot since 1991. The Milwaukee brace, Boston brace, Charleston bending brace, Wilmington brace, Cheneau brace, and Thoraco-Lumbo-Sacral Orthosis have been the most commonly used orthoses in clinical practice and research, with the Milwaukee brace maintaining the longest heat. Since 1995, more clinicians have been concerned about treatment outcomes after a period of follow-up, and interest in the shape and alteration of the rib cage after orthotic treatment has gradually increased. At one point, research has focused on adherence to the SRS criteria. From 2018 onwards, the research focus shifted in several directions: some were about treatment outcomes and assessment parameters, such as quality of life, Cobb angle, success rate, and curve progression; some were related to new treatment schemes, such as combinations with exercises. Several studies have suggested that combining orthoses with exercise can result in better corrective effects.^[30,31]^ There are also other emerging research interests, such as a new 3-dimensional classification of AIS,^[32]^ the validation of some new classifications,^[33,34]^ assessment methods, and finite element models of the scoliotic spine.^[35–38]^

Besides, based on the analysis of co-cited references, the article entitled “Effects of bracing in adolescents with idiopathic scoliosis” contributed by Weinstein in 2013, has been cited the most times. In this study, differences in curve progression, quality of life, and adverse effects between the observation and orthotic treatment groups and the orthotic dose-response relationship were observed. These results indicate that spinal orthoses can significantly reduce the progression of curves and lower the possibility of surgery in patients with AIS.^[5]^ The findings of this research have aroused great interest in follow-up studies, and many clinicians and researchers have begun to conduct research in this direction, taking this study as the most representative work to prove the effectiveness of orthotic treatment for AIS. “The objective measurement of spinal orthosis use for the treatment of adolescent idiopathic scoliosis” of Nicholson published in 2003 and “A prospective study of brace treatment versus observation alone in AIS: a follow-up mean of 16 years after maturity” published by Danielsson in 2007 were the top 2 cited references with the highest centrality. In Nicholson study, data loggers were installed in spinal orthoses to monitor wearing compliance by recording the temperature at the skin-orthosis interface.^[25]^ This study provides an objective method for measuring orthosis compliance and promotes subsequent research on orthosis compliance. The long-term effect of orthoses was investigated in a study by Danielsson in 2017, who came to a similar conclusion to that of Weinstein, that orthotic treatment could depress curve progression and decrease the rate of surgery even after maturity.^[17]^ This study lays a good foundation for research on the long-term effects of orthoses.

The design of orthoses has also been a focus of research in this field. Labelle used a computer-aided tool to assist in the design and adjustment of spinal orthoses and proved its effectiveness in achieving 3-dimensional correction of scoliotic curves.^[39]^ A previous study confirmed the effectiveness of an automated pressure-adjustable orthosis,^[40]^ and a randomized controlled trial was conducted to determine whether the combination of the finite element model and computer aided design and manufacturing method could design more efficient orthoses.^[41]^ Another research topic is the assessment of scoliosis. Researchers have attempted to observe the reliability of a novel assessment method, the ultrasound imaging system,^[42]^ a newly developed computational approach for estimating the plane of maximum curvature has also been proposed,^[43]^ and the cross-cultural adaptation of some assessment questionnaires has been researched.^[38]^

In summary, the effectiveness of orthotic treatment has always been at the frontier of research in this field, particularly in terms of influential factors and outcome indicators. Research on advanced technologies related to orthosis design, such as the finite element model, automated pressure control, and computer algorithms, can promote the development of spinal orthoses and better meet the demands of patients with AIS.

### 4.3. Limitations of the research

This study had some limitations. First, only literature in the core database of the WOS was searched, which may have resulted in a lack of high-quality studies from other databases in the same field. Second, although the bibliometric analysis was executed objectively using the software, the results for the summary of research hotspots were interpreted with an inherent subjective deviation. Furthermore, although the research subject terms were limited in the retrieval process, this did not guarantee that each publication was completely relevant to the core topic. However, the overall status and general trends in the field of orthotic treatment for AIS can still be depicted using the findings of this review.

Future research should concentrate more on 3-dimensional assessments and advanced technologies, such as finite element models and computer algorithms, to improve the treatment outcomes of AIS. It is also suggested to pay more attention to both the functional and psychological needs of the patients, strengthen multidisciplinary communication and cooperation, and promote spinal orthoses to better treat the patients with AIS.

## 5. Conclusions

This bibliometric review and visual analysis showed that research studies on orthotic treatment of AIS has been increased with time. The efficacy and effectiveness of orthotic treatment has consistently been of particular interest. Recent research trends have focused on examining the impact of orthotic treatment on the quality of life, success rates of orthotic treatment, curve progression during bracing, new classification systems for scoliosis, and the effect of exercises. Promising advancements that can be implemented in this field include the use of computer algorithms, innovative designs for spinal orthoses, and pressure control systems.

## Author contributions

**Conceptualization:** Changliang Luo, Huidong Wu, Mansang Wong.

**Data curation:** Changliang Luo, Wei Liu.

**Methodology:** Changliang Luo, Mansang Wong.

**Software:** Changliang Luo, Wei Liu.

**Supervision:** Mansang Wong.

**Visualization:** Huidong Wu.

**Writing – original draft:** Changliang Luo, Wei Liu.

**Writing – review & editing:** Huidong Wu, Mansang Wong.

## References

[1] Hresko MT. Clinical practice. Idiopathic scoliosis in adolescents. N Engl J Med. 2013;368:834–41. 23445094 10.1056/NEJMcp1209063

[2] Grivas TB Burwell GR Vasiliadis ES. A segmental radiological study of the spine and rib—cage in children with progressive infantile idiopathic scoliosis. Scoliosis. 2006;1:17–17. 17049098 10.1186/1748-7161-1-17 PMC1635062

[3] Weinstein SL Dolan LA Cheng JC. Adolescent idiopathic scoliosis. Lancet (London, England). 2008;371:1527–37. 18456103 10.1016/S0140-6736(08)60658-3

[4] Weiss H-R Negrini S Rigo M. Indications for conservative management of scoliosis (guidelines). Scoliosis. 2006;1:5–5. 16759357 10.1186/1748-7161-1-5 PMC1479370

[5] Weinstein SL Dolan LA Wright JG. Effects of bracing in adolescents with idiopathic scoliosis. N Engl J Med. 2013;369:1512–21. 24047455 10.1056/NEJMoa1307337 PMC3913566

[6] Chen B Shin S Wu M. Visualizing the knowledge domain in health education: a scientometric analysis based on CiteSpace. Int J Environ Res Public Health. 2022;19:6440. 35682025 10.3390/ijerph19116440 PMC9180308

[7] Chen C. CiteSpace II: detecting and visualizing emerging trends and transient patterns in scientific literature. J Am Soc Inf Sci Technol. 2006;57:359–77.

[8] Chen C Dubin R Kim MC. Emerging trends and new developments in regenerative medicine: a scientometric update (2000–2014). Expert Opin Biol Ther. 2014;14:1295–317. 25077605 10.1517/14712598.2014.920813

[9] Sun W Song J Dong X. Bibliometric and visual analysis of transcranial direct current stimulation in the web of science database from 2000 to 2022 via CiteSpace. Front Hum Neurosci. 2022;16:1049572–1049572. 36530203 10.3389/fnhum.2022.1049572 PMC9751488

[10] Wu H Zhou Y Xu L. Mapping knowledge structure and research frontiers of ultrasound-induced blood-brain barrier opening: a scientometric study. Front Neurosci. 2021;15:706105–706105. 34335175 10.3389/fnins.2021.706105 PMC8316975

[11] Zhu K Lin R Li H. Study of virtual reality for mild cognitive impairment: a bibliometric analysis using CiteSpace. Int J Nurs Sci. 2022;9:129–36. 35079614 10.1016/j.ijnss.2021.12.007 PMC8766785

[12] Newman MEJ. Modularity and community structure in networks. Proc Natl Acad Sci USA. 2006;103:8577–8582. From the Cover. doi:10.1073/pnas.0601602103. 16723398 10.1073/pnas.0601602103 PMC1482622

[13] Zhou Q Kong H-B He B-M. Bibliometric analysis of bronchopulmonary dysplasia in extremely premature infants in the web of science database using CiteSpace software. Front Pediatr. 2021;9:705033–705033. 34490163 10.3389/fped.2021.705033 PMC8417835

[14] Lin G-X Chen C-M Rui G. Research relating to three-dimensional (3D) printing in spine surgery: a bibliometric analysis. Eur Spine J. 2023;32:395–407. 36109389 10.1007/s00586-022-07376-8

[15] Pei W Peng R Gu Y. Research trends of acupuncture therapy on insomnia in two decades (from 1999 to 2018):a bibliometric analysis. BMC Complement Altern Med. 2019;19:225–225. 31438914 10.1186/s12906-019-2606-5 PMC6704508

[16] Butt NS Malik AA Shahbaz MQ. Bibliometric analysis of statistics journals indexed in web of science under emerging source citation index. SAGE Open. 2021;11:215824402098887.

[17] Danielsson AJ Hasserius R Ohlin A. A prospective study of brace treatment versus observation alone in adolescent idiopathic scoliosis: a follow-up mean of 16 years after maturity. Spine. 2007;32:2198–207. 17873811 10.1097/BRS.0b013e31814b851f

[18] Janicki JA Poe-Kochert C Armstrong DG. A comparison of the thoracolumbosacral orthoses and providence orthosis in the treatment of adolescent idiopathic scoliosis: results using the new SRS inclusion and assessment criteria for bracing studies. J Pediatr Orthop. 2007;27:369–74. 17513954 10.1097/01.bpb.0000271331.71857.9a

[19] Karol LA Virostek D Felton K. Effect of compliance counseling on brace use and success in patients with adolescent idiopathic scoliosis. J Bone Joint Surg Am. 2016;98:9–14. 26738898 10.2106/JBJS.O.00359

[20] Thompson RM Hubbard EW Jo C-H. Brace success is related to curve type in patients with adolescent idiopathic scoliosis. J Bone Joint Surg Am. 2017;99:923–8. 28590377 10.2106/JBJS.16.01050

[21] Negrini S Aulisa AG Aulisa L. 2011 SOSORT guidelines: orthopaedic and rehabilitation treatment of idiopathic scoliosis during growth. Scoliosis. 2012;7:3–3. 22264320 10.1186/1748-7161-7-3 PMC3292965

[22] Negrini S Donzelli S Aulisa AG. 2016 SOSORT guidelines: orthopaedic and rehabilitation treatment of idiopathic scoliosis during growth. Scoliosis Spinal Dis. 2018;13:3–3. 10.1186/s13013-017-0145-8 PMC5795289 29435499

[23] Qiu Y Sun X Cheng JCY. Bone mineral accrual in osteopenic and non-osteopenic girls with idiopathic scoliosis during bracing treatment. Spine. 2008;33:1682–9. 18594461 10.1097/BRS.0b013e31817b5b9e

[24] Havey R Gavin T Patwardhan A. A reliable and accurate method for measuring orthosis wearing time. Spine. 2002;27:211–4. 11805670 10.1097/00007632-200201150-00018

[25] Nicholson GP Ferguson-Pell MW Smith K. The objective measurement of spinal orthosis use for the treatment of adolescent idiopathic scoliosis. Spine. 2003;28:2243–50; discussion 2250. 14520038 10.1097/01.BRS.0000085098.69522.52

[26] Aulisa AG Galli M Giordano M. Initial in-brace correction: can the evaluation of cobb angle be the only parameter predictive of the outcome of brace treatment in patients with adolescent idiopathic scoliosis? Children (Basel). 2022;9:338. 35327710 10.3390/children9030338 PMC8946904

[27] Sato M Ohashi M Tashi H. Association of success of brace treatment and various aspects of in-brace correction in patients with adolescent idiopathic scoliosis. J Orthop Sci. 2023;28:1221–6. 36372677 10.1016/j.jos.2022.10.001

[28] Peeters CMM van Hasselt AJ Wapstra FH. Predictive factors on initial in-brace correction in idiopathic scoliosis: a systematic review. Spine. 2022;47:E353–61. 35500086 10.1097/BRS.0000000000004305

[29] Meng Z-D Li T-P Xie X-H. Quality of life in adolescent patients with idiopathic scoliosis after brace treatment: a meta-analysis. Medicine (Baltim). 2017;96:e6828–e6828. 10.1097/MD.0000000000006828 PMC5428595 28489761

[30] da Silveira GE Andrade RM Guilhermino GG. The effects of short- and long-term spinal brace use with and without exercise on spine, balance, and gait in adolescents with idiopathic scoliosis. Medicina (Kaunas, Lithuania). 2022;58:1024. 36013490 10.3390/medicina58081024 PMC9413676

[31] Larni Y Mohsenifar H Ghandhari H. The effectiveness of Schroth exercises added to the brace on the postural control of adolescents with idiopathic scoliosis: case series. Ann Med Surg (2012). 2022;84:104893–104893. 10.1016/j.amsu.2022.104893 PMC9758328 36536721

[32] Shen J Parent S Wu J. Towards a new 3D classification for adolescent idiopathic scoliosis. Spine Deform. 2020;8:387–96. 32026444 10.1007/s43390-020-00051-2

[33] Fruergaard S Jain MJ Deveza L. Evaluation of a new sagittal classification system in adolescent idiopathic scoliosis. Eur Spine J. 2020;29:744–53. 31802239 10.1007/s00586-019-06241-5

[34] Lin JD Osorio JA Baum GR. A new modular radiographic classification of adult idiopathic scoliosis as an extension of the Lenke classification of adolescent idiopathic scoliosis. Spine Deform. 2021;9:175–83. 32748229 10.1007/s43390-020-00181-7

[35] Jafarian F-S Yeowell G Sadeghi-Demneh E. Cross-cultural adaptation and validation of the Bad Sobernheim Stress questionnaire in Iranian adolescents with idiopathic scoliosis using thoracolumbar orthoses. Adv Biomed Res. 2022;11:39–39. 35814302 10.4103/abr.abr_154_21 PMC9259448

[36] Park SH Goh TS Park YG. Validation of a Korean version of the quality-of-life profile for spine deformities (QLPSD) in patients with adolescent idiopathic scoliosis. Eur Rev Med Pharmacol Sci. 2022;26:84–9. 10.26355/eurrev_202201_27751 35049023

[37] Vergari C Chen Z Robichon L. Towards a predictive simulation of brace action in adolescent idiopathic scoliosis. Comput Methods Biomech Biomed Engin. 2021;24:874–82. 33295806 10.1080/10255842.2020.1856373

[38] Yi H Chen H Wang X. Cross-cultural adaptation and validation of the Chinese version of the brace questionnaire. Front Pediatr. 2021;9:763811–763811. 35096702 10.3389/fped.2021.763811 PMC8793733

[39] Labelle H Bellefleur C Joncas J. Preliminary evaluation of a computer-assisted tool for the design and adjustment of braces in idiopathic scoliosis: a prospective and randomized study. Spine (Philadelphia, Pa 1976). 2007;32:835–43. 10.1097/01.brs.0000259811.58372.87 17426626

[40] Lin Y Lou E Lam TP. The intelligent automated pressure adjustable orthosis for patients with adolescent idiopathic scoliosis: a bi-center randomized controlled trial. Spine (Phila Pa 1976). 2020;45:1395–402. 32453223 10.1097/BRS.0000000000003559

[41] Cobetto N Aubin C-E Parent S. 3D correction of AIS in braces designed using CAD/CAM and FEM: a randomized controlled trial. Scoliosis. 2017;12:24–24. 10.1186/s13013-017-0128-9 PMC5525241 28770254

[42] Wang Q Li M Lou EHM. Reliability and validity study of clinical ultrasound imaging on lateral curvature of adolescent idiopathic scoliosis. PloS one. 2015;10:e0135264. 26266802 10.1371/journal.pone.0135264 PMC4534204

[43] Wu H-D He C Chu WC-W. Estimation of plane of maximum curvature for the patients with adolescent idiopathic scoliosis via a purpose-design computational method. Eur Spine J. 2021;30:668–75. 32767126 10.1007/s00586-020-06557-7

